# Flow Control Based on Feature Extraction in Continuous Casting Process

**DOI:** 10.3390/s20236880

**Published:** 2020-12-01

**Authors:** Shereen Abouelazayem, Ivan Glavinić, Thomas Wondrak, Jaroslav Hlava

**Affiliations:** 1 Faculty of Mechatronics, Informatics and Interdisciplinary Studies, Technical University of Liberec, 461 17 Liberec, Czech Republic; jaroslav.hlava@tul.cz; 2 Helmholtz-Zentrum Dresden—Rossendorf, 01328 Dresden, Germany; i.glavinic@hzdr.de (I.G.); t.wondrak@hzdr.de (T.W.)

**Keywords:** industrial control, industrial process tomography, model predictive control, ultrasound doppler velocimetry

## Abstract

The flow structure in the mold of a continuous steel caster has a significant impact on the quality of the final product. Conventional sensors used in industry are limited to measuring single variables such as the mold level. These measurements give very indirect information about the flow structure. For this reason, designing control loops to optimize the flow is a huge challenge. A solution for this is to apply non-invasive sensors such as tomographic sensors that are able to visualize the flow structure in the opaque liquid metal and obtain information about the flow structure in the mold. In this paper, ultrasound Doppler velocimetry (UDV) is used to obtain key features of the flow. The preprocessing of the UDV data and feature extraction techniques are described in detail. The extracted flow features are used as the basis for real time feedback control. The model predictive control (MPC) technique is applied, and the results show that the controller is able to achieve optimum flow structures in the mold. The two main actuators that are used by the controller are the electromagnetic brake and the stopper rod. The experiments included in this study were obtained from a laboratory model of a continuous caster located at the Helmholtz-Zentrum Dresden Rossendorf (HZDR).

## 1. Introduction

**Figure 1:**
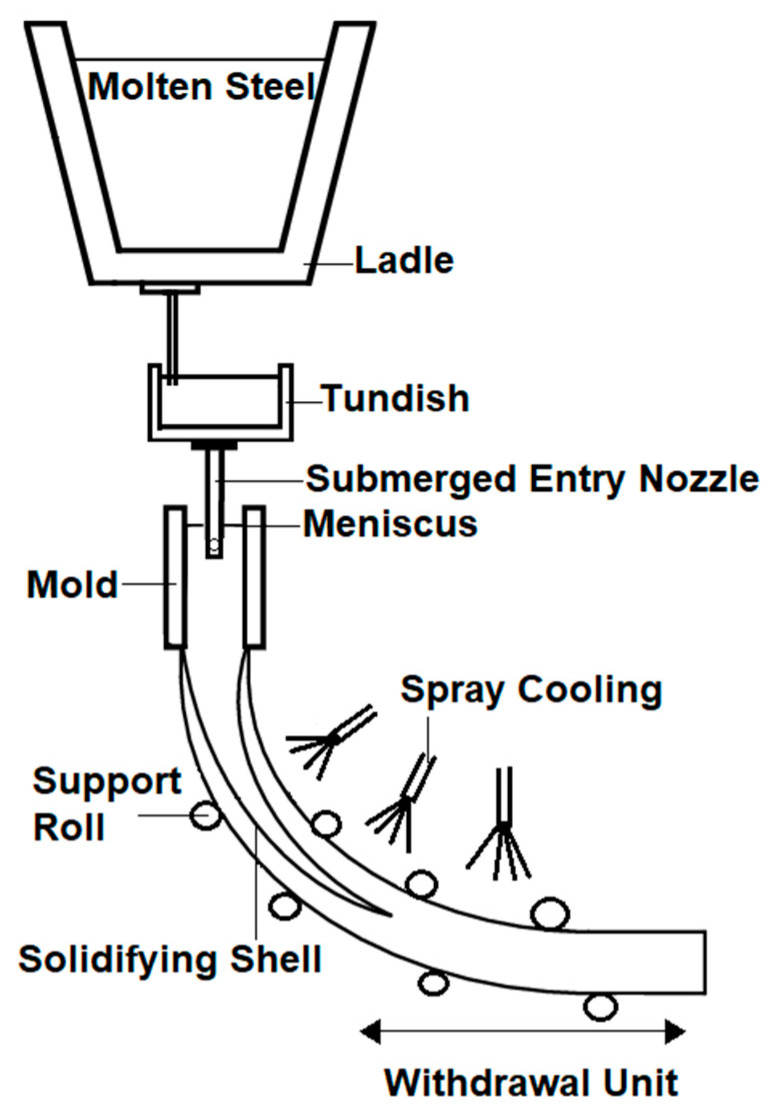
Schematic of a continuous casting process.

The majority of the world’s steel production uses continuous casting to produce solid steel. Although the process has been around for many decades, the complex flow phenomena especially in the mold of a continuous caster are still being investigated and modelled. The continuous casting process is shown in Figure 1; liquid steel is first poured from a ladle into a tundish, then it flows into the mold through a submerged entry nozzle (SEN). The flow rate in the SEN is controlled by either a stopper rod or a sliding gate. The liquid steel begins to cool down and takes the shape of the mold. This creates a strand that is pulled out by water cooled rollers until the solidification process is fully completed [1]. Phenomena including clogging, turbulent flow, and slag entrapment are heavily studied in the industry due to their detrimental effects on the quality of the steel [2]. Many of these quality issues originate from the flow structure of the liquid steel in the mold. The visualization of the flow in this area is very difficult due to limitations of sensors. This limitation presents also a challenge for controller designs that could potentially improve the quality issues in the mold. For this reason, most of the previous research in this area rely on indirect measurements. The flow is neither directly measured nor directly controlled but the control takes only mold level fluctuations as input into account. The main control objective is keeping the mold level as constant as possible. Examples are presented in a paper by Kim et al. [3], in which the authors attempt to control the mold level using an adaptive fuzzy controller. The controller tries to decrease the bulging disturbance effect that is usually seen in molten steel level. The performance of the controller was tested on a hardware simulator which showed that the bulging effect was reduced. A similar attempt achieving constant mold level was the use of a proportional–integral–derivative (PID) controller with an additional disturbance observer and feedforward compensation [4].

**Figure 2:**
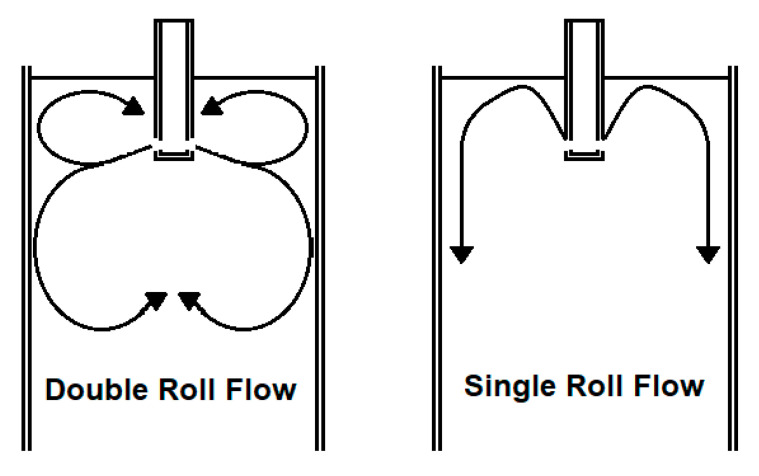
Flow patterns in a mold.

Another main research topic is the application of electromagnetic actuators to directly influence the flow field in the mold. An electromagnetic brake (EMBr) generates a static magnetic field, which is used to influence the flow in specific regions of the mold [5]. In numerical simulations and plant trials it was shown that the electromagnetic brake is able to decrease the internal defects in the produced steel [6,7]. Further research indicates that the position of the brake relative to the submerged entry nozzle in the mold can significantly change the mold flow [8]. Additionally, electromagnetic stirrers use an alternating magnetic field to stir the molten steel in the mold. In a paper by Maurya et al. [9], the authors use finite element method to simulate the effect of different vertical positions of the stirrer on the flow and solidification in the mold. However, actuation is only one part of the control loop. It is necessary to know the objectives of the control, i.e., which flow pattern is the most desirable one. Finally, the real time information about flow pattern has to be known, to control the actuators effectively. Previous investigations show that the optimum flow pattern in a slab mold is a double roll flow rather than a single roll flow as seen in Figure 2. The double roll flow promotes the flotation of non-metallic inclusions to the meniscus and minimizes mold level fluctuations, slag entrainment, and other surface defects [10,11].

Several approaches were proposed to obtain information about flow characteristics. Zhang et al. [11] uses a rod deflection method connected with complex mathematical model that combines computational fluid dynamics and discrete phase method. Hashimoto et al. [12] develops a real-time flow estimation algorithm based on a three-dimensional transient modeling in order to obtain information on the steel flow. However, this approach was validated by simulations only. In the last few years the monitoring of the two-dimensional temperature distribution in the wide faces of the mold using optical fiber Bragg gratings was developed [13]. However, the estimation of the flow structure based on the temperature measurements is still challenging.

In this paper, we propose the use of tomographic sensors to obtain information about the full structure of the flow in the mold rather than specific point variables such as the mold level. Ultrasound Doppler velocimetry (UDV) will be used as a substitute for contactless inductive flow tomography (CIFT) and the results will be analyzed similarly to a paper by Choi et al. [14] where the authors compare the velocity profiles obtained by UDV and magnetic resonance imagining (MRI) in fluid foods. Moving along, flow control in the mold will be achieved using model predictive control to reach the optimum flow required. Using the velocity profile measured by UDV, we can visualize the flow structure in the mold. This will allow us to extract important features of the flow that will be utilized by the controller. It should be noted that the described techniques to design the control loop can be utilized for other tomographic sensors as well, assuming that sufficient information on the velocity profile of the mold is obtained. Furthermore, the work presented in this paper is based on previous papers [15,16] where the authors have used UDV measurements to create preliminary control loops to control either the angle of the jet or the meniscus velocity. The presented concepts will be extended to demonstrate that the flow structure obtained by UDV can be fully utilized for control.

## 2. Experimental Setup

### 2.1. Mini-LIMMCAST Facility

**Figure 3:**
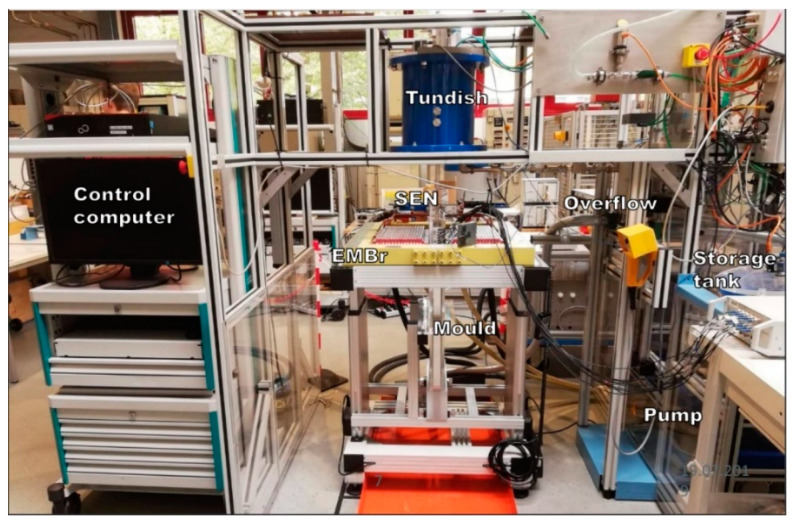
Mini-LIMMCAST Facility in HZDR.

The experiments included in this study were conducted with the Mini-LIMMCAST facility located at Helmholtz-Zentrum Dresden-Rossendorf (HZDR) which is shown in Figure 3.

**Figure 4:**
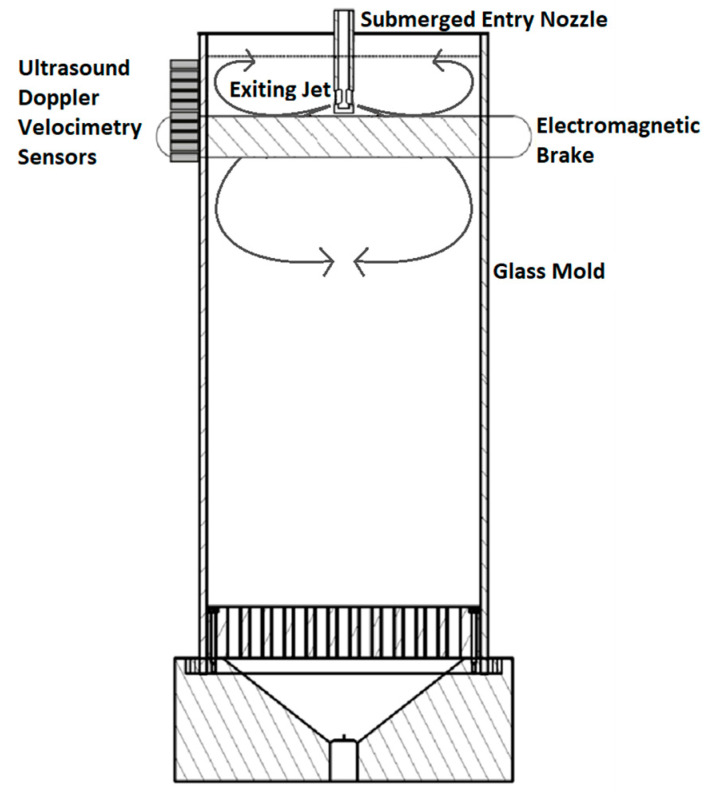
Schematic of mold illustrating the position of the sensors and electromagnetic brake.

The setup consists of a small-scale model of the mold and strand of a continuous slab caster. Gallium-indium-tin (GaInSn) eutectic alloy is used in place of the liquid steel because it is a liquid at room temperature. GaInSn is poured from the ’tundish’ into an acrylic glass mold via the SEN as shown in Figure 4. The flow rate in the SEN is controlled by a stopper rod. In order to run the experiments continuously, the melt flows from the bottom of the mold into the storage vessel, from which liquid is pumped back to the tundish to repeat the process. The physical properties and dimensions of the setup can be found in Table 1 and Table 2. An electromagnetic brake is used to alter the flow in the mold. This will be discussed in more detail in the next sections. UDV is used during the experiments to reconstruct the flow in the mold by measuring the horizontal velocity component of the flow in the opaque liquid.

Figure 4 shows the 10 UDV sensors placed vertically on the narrow face of the mold with 10 mm spacing in between. Each UDV sensor measures the velocity of small moving particles in GaInSn and returns the velocity profile along the horizontal ultrasound beam. The measurements were conducted using the DOP3000 which is able to operate 10 transducers [18]. The transmitters are activated sequentially and this cycle is repeated so that the flow in the left half region of the mold is visualized.

### 2.2. Electromagnetic Brake

The electromagnetic brake will be one out of two manipulated variables in the control loop. Generally, in continuous casting electromagnetic brakes and stirrers are used to stabilize the turbulent flow in the mold. However, due to the limitations of using conventional sensors during the process, the electromagnetic actuators are applied at specific pre-calculated strengths. Currently, there is a very limited number of investigations on using real-time control of the electromagnetic actuators. To our knowledge there is only one paper published by Dekemele et al. [18] where the authors use an electromagnetic stirrer to keep the meniscus flow within an optimum range. A static model and a dynamic model were identified using system identification. Both PI and a specific MPC method called extended prediction self-adaptive control (EPSAC) are used to achieve the desired velocity. The closed loop simulations show that the EPSAC performs far better than the PI, however it requires more aggressive control effort.

In the Mini-LIMMCAST setup a static single ruler brake is used. Under the influence of the static magnetic field the velocity of the melt drives an electrical current in the melt. The current, in turn, generates the Lorentz force acting on the melt. It is assumed that the force is damping the velocities. The placement of the electromagnetic brake is critical for the control design as it has been shown that small variations in the vertical position of the brakes can significantly change the effects of the brake on the flow [18]. For the experiments, the optimal position for the electromagnetic brake is chosen where the pole shoes are right below the SEN.

By analyzing the measured velocity fields gathered by UDV, it became clear that the brake moves the jet exiting the SEN upward so that it becomes more horizontal. In the experiments, the current of the brake will be varied from 0 to 600 A. This will allow us to create a model describing the relationship between the flow structure in the mold and the current using system identification. This will later be used as the basis for the controller design.

## 3. Pre-Processing Data for Control

The main two actuators used by the controller to influence the flow of the mold are the electromagnetic brake and the stopper rod in the SEN. In order to design the controller and test its performance, a model describing the relationship between the manipulated variables and the controlled variables is needed. There is extensive research on the modelling of the continuous casting process using computational fluid dynamics [2,19], where a combination of Navier-Stokes equation with Lorentz force as seen in Equations (1)–(4) are solved by discretizing the mold and using boundary conditions in order to achieve a system of algebraic equations [19]:

(1) $$\nabla \cdot \vec{u}=0$$

(2) $$\frac{\partial \vec{u}}{\partial t}+\nabla \cdot \left(\vec{u}\times \vec{u}\right)=-\frac{1}{\rho }\vec{\nabla }p+\nabla \cdot \tau_{lam}-\nabla \cdot \tau_{SGS}+\frac{1}{\rho }{\vec{J}}_{F}\times {\vec{B}}_{0}$$

(3) $${\vec{J}}_{F}=\sigma \left(\vec{u}\times {\vec{B}}_{0}-\vec{\nabla }\phi \right)$$

(4) $$\nabla \cdot \vec{\nabla }\phi =\nabla \cdot \left(\vec{u}\times {\vec{B}}_{0}\right)$$

In the above equations, $\tau_{lam}$ represents the laminar viscous stress and $\tau_{SGS}$ represents the stress tensor which is evaluated using a turbulence model. In order to model the effect of the electromagnetic brake on the velocity fields of the mold, the Lorentz force term is added in the momentum equation as the cross product between the current density in the fluid ${\vec{J}}_{F}$ and the applied magnetic field ${\vec{B}}_{0}$, where ϕ represents the electric potential. Although this methodology has been successful applied simulating the flow behavior in the mold, there are many challenges when it comes to utilizing these models for model-based control. The complex geometry of the submerged entry nozzle and the high turbulence in the mold require a fine mesh to solve the algebraic equations. An example can be seen in [19] where a mesh of around 2.7 million cells is needed to simulate the process. This severely limits the possibility of using this approach for designing a control loop as the calculation of the flow has to be achieved in real time, which becomes complicated with models of this order. Another approach used to create the necessary models for simulating and designing the controller is system identification, where a black box model is created using experimental data. This allows for simpler models to be created that are able to efficiently describe the dependence of the flow field in the mold from the position of the stopper rod and the strength of the brake. This methodology will be discussed in more depth in later sections.

### 3.1. Pre-Processing of the UDV Data

**Figure 5:**
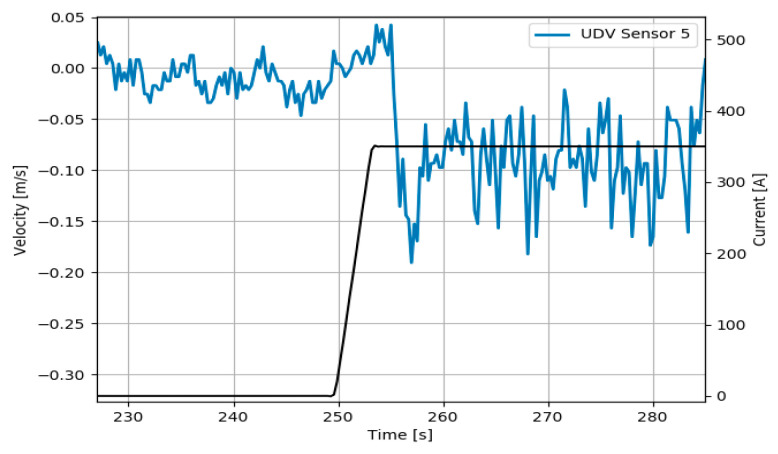
Dynamic velocity response to step increase in current from 0 A to 350 A [16].

**Figure 6:**
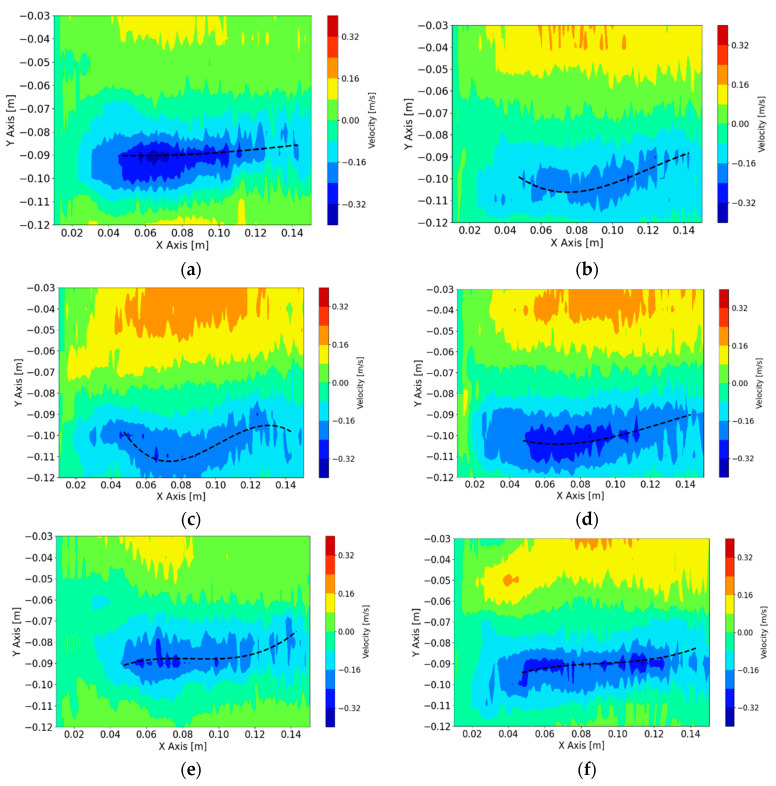
Reconstruction of velocity profile with tracking of jet shape to quantify jet velocity, (**a**) *t* = 200 s, (**b**) *t* = 400 s, (**c**) *t* = 500 s, (**d**) *t* = 600 s, (**e**) *t* = 800 s, (**f**) *t* = 1050 s.

The turbulent nature of the flow in the mold is clearly seen in the data measured by the UDV sensor 5 given in Figure 5. In the steady state time intervals before 250 s and after 260 s, the signal appears noisy due to the turbulent nature of the liquid in the mold. A median filter is used to dampen the noise while preserving the fast-dynamic changes that occur from changing the manipulated variable as seen at *t* = 255 s. Furthermore, the general flow structure in the left region of the mold can be reconstructed by plotting the velocity profile as shown in Figure 6. The UDV sensors are placed with a gap of 10 mm between each sensor. In order to achieve a finer resolution in the vertical axis a 1-D cubic spline interpolation is used. As seen in Figure 6, the negative velocities indicate the flow moving towards the narrow face (towards the sensors), while the positive velocities indicate flow moving away from the narrow face. In these figures the shape and velocity of the exiting jet from the SEN can be clearly seen.

### 3.2. Feature Extraction

In order to avoid using all velocity values in the region, the concept of feature extraction is used to extract the specific features of the flow that are the most useful for indicating whether the flow is optimal or not. Similar concepts are presented in a paper by Xie et al. [20] where the gray level of pixels in specific regions of the images produced by electrical capacitance tomography (ECT) are used to distinguish between flow patterns in a two-phase flow. Another example is given by Bukhari et al. [21] where ECT was applied on an oil separator. The percentages of water, oil, and air were obtained by applying principal component analysis (PCA) on the pixels of the ECT image. Euclidean distance was then used to compare the image to data sets in a knowledge base to determine which control action is appropriate in real time.

For the case of the continuous caster, we need to determine the specific features of the flow in the mold that affect the quality of the steel. The two features chosen in this paper for control are the jet impingement point on the narrow face, and the velocity of the exiting jet. The importance of the jet impingement point is analyzed by Zhang et al. [22] where a deeper impingement into the mold correlated with higher slag entrapment and argon bubbles being trapped deep in the mold. Furthermore, the importance of the velocity of the exiting jet is shown by Thomas [23,24] where the strength of the jet can affect the steel shell at the impingement point on the narrow face where it impinges. Additionally, the strength of the jet influences the shape of the meniscus.

**Figure 7:**
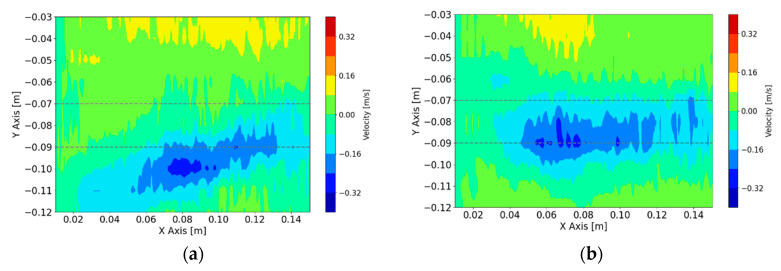
Reconstruction of velocity profile with identified shallow region to quantify jet impingement (**a**) *t* = 300 s, (**b**) *t* = 800 s.

The jet impingement point defines how deep or shallow the jet impinges into the mold. The optimum case is to keep the jet as close to the horizontal baseline as possible to ensure a shallow impingement. As shown in Figure 7, this feature can be quantified by calculating the mean value of the velocity field between UDV sensors 5 to 7 (−0.07 m to −0.09 m from the surface level). If the value increases, it is more likely that the exiting jet is oscillating in this region. The value of the mean velocity will be used as the controlled variable.

The idea of using the velocity of the exiting jet is based on previous work [16] where a straight line was used to represent the exiting jet. The shape of the jet can be identified by scanning for every vertical line in the velocity map for the largest negative velocity and fitting a line using linear regression which would then represent the exiting jet. In this paper, we will be extending this concept to include a more realistic shape of the jet, which has sometimes a more ‘banana’ like shape. In order to model this adequately, a third-degree polynomial is used to fit the shape of the jet during each captured frame as shown in Figure 6. It is clear that the polynomial can track the movement and shape of the jet efficiently. The controlled variable is the mean of the velocities along the polynomial.

### 3.3. System Identification

A black-box model is created based on both extracted features from the UDV measurements and applying system identification to determine the dynamic relationship between the inputs and outputs. A 2-input, 2-output model is created where the inputs are the current of electromagnetic brake and the stopper rod position, while the outputs are the jet impingement point and the jet velocity. The current of the brake was varied from 0 to 600 A, while the lifting of the stopper rod position was between 5–10 mm.

**Figure 8:**
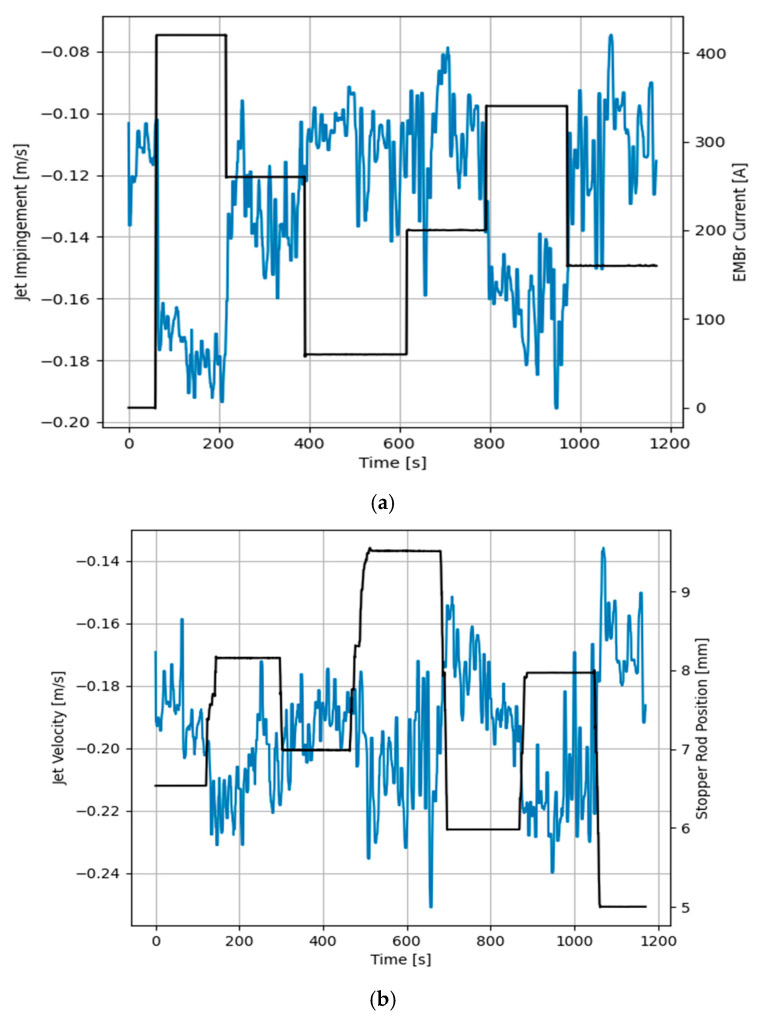
Measured response of jet impingement to manipulated variables, (**a**) Electromagnetic brake current, (**b**) Stopper rod position.

**Figure 9:**
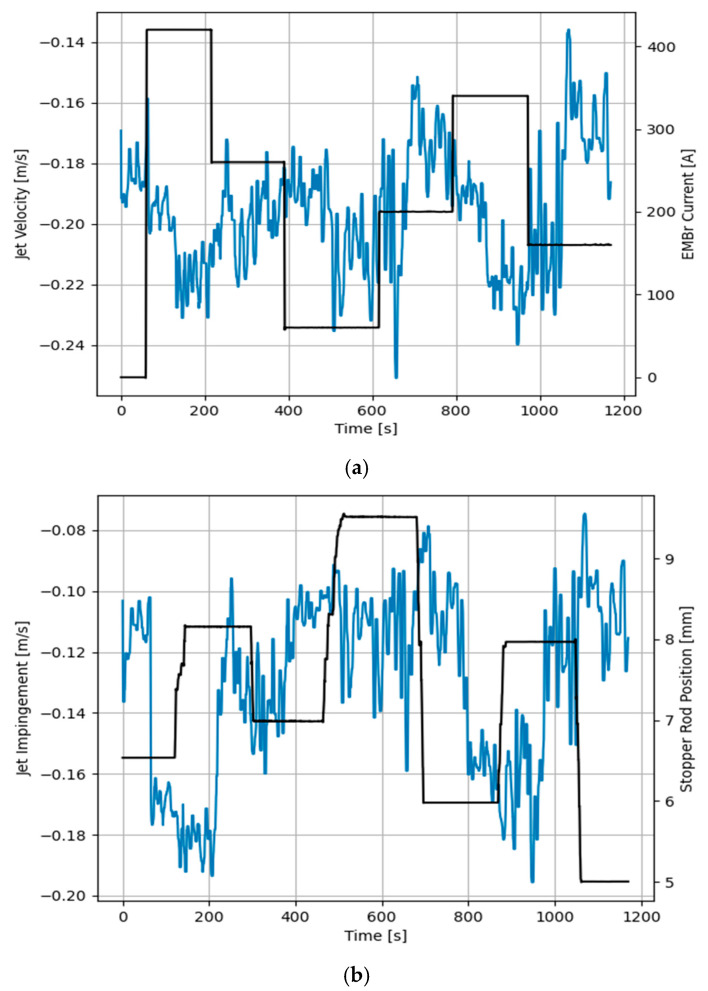
Measured response of jet velocity to both manipulated variables, (**a**) Electromagnetic brake current, (**b**) Stopper rod position.

As seen in Figure 8 and Figure 9, random input steps are applied to both the electromagnetic brake and stopper rod position to excite the full dynamics of the process. It becomes clear that both features have fast dynamic responses to the manipulated variables. Figure 8a shows that increasing the current of the brake significantly increases the jet impingement value which correlates to a shallow impingement point. Furthermore, increasing the current of the brake increases the jet velocity. However, this effect is less significant. On the other side, increasing the stopper rod position also lifts the impingement point, as shown in Figure 8b, but the effect is less significant in comparison with the strength of the brake, while the effect is more pronounced for the jet velocity.

State space estimation was applied to create a 4th order discrete state space model using subspace method in the form:

(5) $$\dot{x}\left(kT+T\right)=Ax\left(kT\right)+Bu\left(kT\right)+Ke\left(kT\right)$$

**Figure 10:**
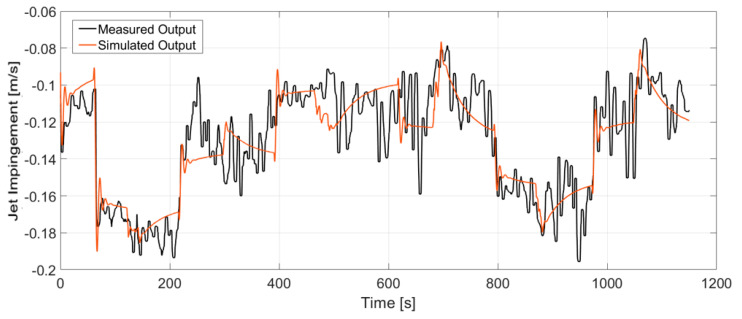
Comparison of the output of identified model with the measured output for jet impingement. The deterministic part of the output relevant for model-based control is captured sufficiently by the model.

**Figure 11:**
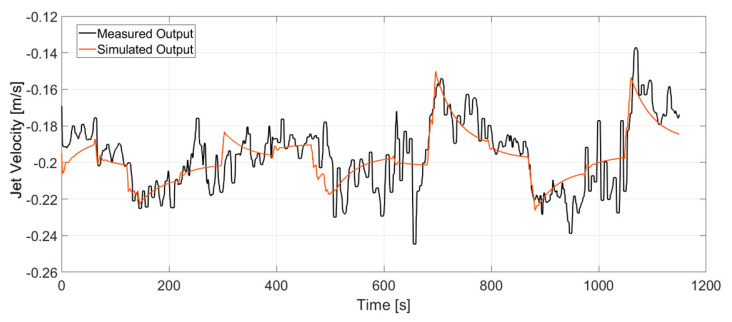
Comparison of the output of identified model with the measured output for jet velocity. The deterministic part of the output relevant for model-based control is captured sufficiently by the model.

(6) y(kT)=Cx(kT)+Du(kT)+e(kT)

where *A*, *B*, *C*, *D* and *K* are the state-space matrices. Disturbance component *K* is set to 0, while the sample time *T* = 0.3 s. *u(kT)* represents the input to the system which are the current of the electromagnetic brake and position of the stopper rod, while *y(kT)* represents the output which are the jet impingement and velocity. The output of the state space model was compared with the measurement output in Figure 10 and Figure 11. It is clear that the model is able to track the deterministic part of the signal which is the dynamic response due to the changes to the manipulated variables. The stochastic part of the signal that is due to the turbulent nature of the flow [2] is not described by the model. However it turned out that we do not need to describe this part of the signal to build the model-based controller.

**Figure 12:**
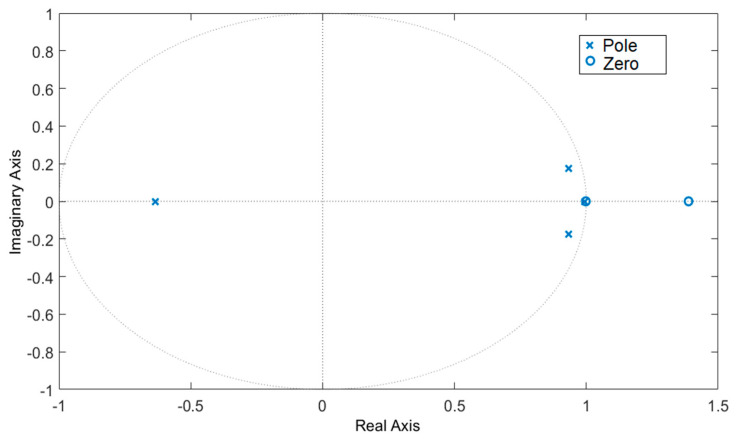
Pole-Zero plot for identified discrete model indicating a non-minimum phase system.

By analyzing the pole-zero plot of the discrete system it becomes clear that we are dealing with a non-minimum phase system due to zeros outside the unit circle as seen in Figure 12. Non-minimum phase systems have inverses that are unstable, this means that the response of the system to a step input will consist of an ‘undershoot’. When a step input is introduced, the output of the system will first change in opposite direction before converging to the final value. This introduces extra complexity when it comes to controlling the system due to issues regarding performance and robustness. One way to tackle this issue is to use predictive control such as Model Predictive Control. In this way the controller can predict the future changes in the output and anticipate this initial change in direction before settling to the steady state value.

## 4. Model Predictive Control

Model Predictive Control has been successfully implemented in many complex industrial applications due to its ability to incorporate constraints in its algorithm. Furthermore, Model Predictive Control algorithms can easily be expanded for multivariable control problems [25,26,27]. The main theory behind this control technique is the iterative, finite-horizon optimization of an internal plant model using a cost function *J* over the receding horizon. In this paper, the cost function consists of the sum of three terms:

(7) $$J\left(z_{k}\right)=J_{y}\left(z_{k}\right)+J_{\Delta u}\left(z_{k}\right)+J_{e}\left(z_{k}\right)$$

where *z_k_* is the vector of the quadratic program decision variables. $J_{y}\left(z_{k}\right)$ refers to the output reference tracking, $J_{\Delta u}\left(z_{k}\right)$ is for the manipulated variable move suppression, and $J_{e}\left(z_{k}\right)$ refers to constraint violations as seen below:

(8) $$J_{y}\left(z_{k}\right)=\overset{n_{y}}{\underset{j=1}{\sum }}\overset{p}{\underset{i=1}{\sum }}{\left\{\frac{w^{y}{}_{i,j}}{s^{y}{}_{j}}\left[r_{j}\left(k+j|k\right)-y_{j}\left(k+j|k\right)\right]\right\}}^{2}$$

(9) $$J_{\Delta u}\left(z_{k}\right)=\overset{n_{u}}{\underset{j=1}{\sum }}\overset{p-1}{\underset{i=0}{\sum }}{\left\{\frac{w^{\Delta u}{}_{i,j}}{s^{u}{}_{j}}\left[u_{j}\left(k+i|k\right)-u_{j}\left(k+j-1|k\right)\right]\right\}}^{2}$$

(10) $$J_{e}\left(z_{k}\right)=\rho_{\varepsilon }\varepsilon_{k}^{2}$$

where *k* is the current control interval, *p* is the prediction horizon, *n_y_* and *n_u_* are the number of plant output variables and number of manipulated variables. $y_{j}\left(k+j|k\right)$ and $r_{j}\left(k+j|k\right)$ are the predicted value and reference value of *j*-th plant output at *i*-th prediction horizon. $s^{y}{}_{j}$ and $s^{u}{}_{j}$ are the scale factor for *j*-th plant output and manipulated variable, respectively. $w^{y}{}_{i,j}$ and $w^{\Delta u}{}_{i,j}$ are the tunning weights for the *j*-th plant output and manipulated variable movement at *i*th prediction horizon. $\varepsilon_{k}$ is the slack variable at control interval *k*. $\rho_{k}$ is the constraint violation penalty weight [28]. The main control objectives for the experiments conducted in this study are to achieve a shallow jet impingement and maintain the jet velocity within an optimum range. The first controlled variable $y_{1}\left(k+i-1\right)$ is the jet impingement, while the second controlled variable $y_{2}\left(k+i-1\right)$ is the jet velocity. The following constraints are applied on the manipulated variables to respect the limitations of both the electromagnetic brake and stopper rod:

(11) $$0\leq u_{1}\left(k+i-1\right)\leq 600$$

(12) $$5\leq u_{2}\left(k+i-1\right)\leq 10$$

(13) $$-100\leq \Delta u_{1}\left(k+i-1\right)\leq 100$$

(14) $$-1\leq \Delta u_{1}\left(k+i-1\right)\leq 1$$

where the first manipulated variable $u_{1}\left(k+i-1\right)$ is the current of the electromagnetic brake, while the second manipulated variable $u_{2}\left(k+i-1\right)$ is the position of the stopper rod. The proposed controller parameters for the MPC are listed in Table 3. In this case, avoiding large increments in the manipulated variables is desirable in order to create a more robust performance from the controller. Although this will compromise the reference tracking, increasing the manipulated variable rate weights will help compensate for the changes in the velocities due to the turbulent flow that is not fully described by the internal model of the controller.

## 5. Results and Discussion

**Figure 13:**
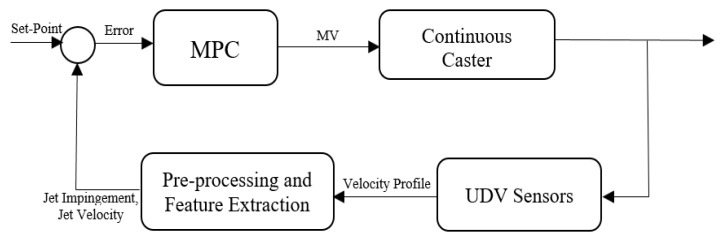
Control loop based on measured velocity profile from ultrasound Doppler velocimetry.

Figure 13 illustrates the control loop implemented in the following experiments. The algorithm starts by processing the raw data from the UDV and constructing the velocity profile in the desired region of the mold. The next step is to extract the necessary features from the velocity profile including the jet impingement and jet velocity. These features can then be processed by the controller where a set-point reference for both controlled variables are implemented. The set-points for the following experiments are the optimal values that were discussed in Section 4. Lastly, based on the error between the set-points and the controlled variables, the controller decides how to change both actuators utilizing the internal prediction model and cost function (Equation (7)).

**Figure 14:**
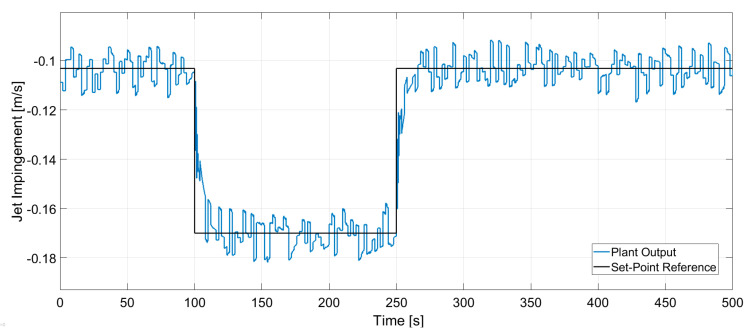
Closed loop response of jet impingement for set-point tracking.

**Figure 15:**
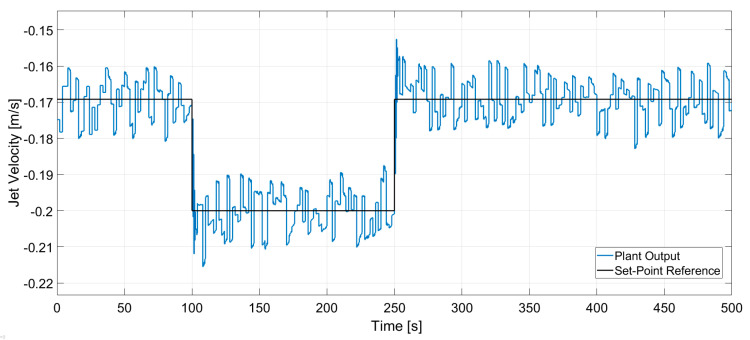
Closed loop response of jet velocity for set-point tracking.

**Figure 16:**
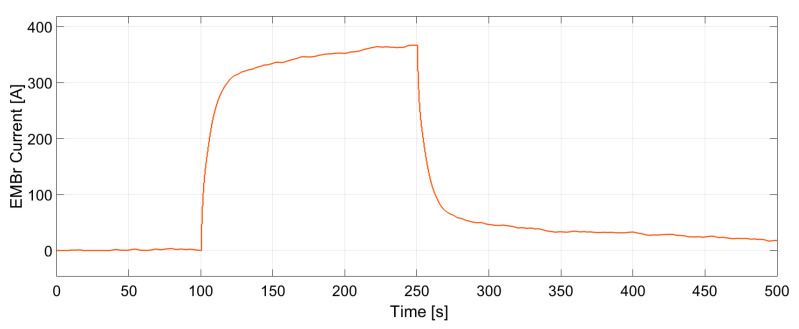
Changes of electromagnetic brake current generated by the controller to track the jet impingement. The manipulated variable does not exceed the constraints of the brake.

**Figure 17:**
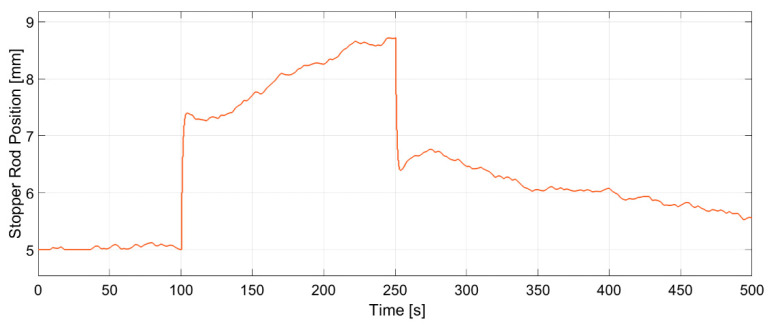
Changes of the stopper rod position generated by the controller to track the jet velocity. The manipulated variable does not exceed the constraints of the stopper rod.

In Figure 14 and Figure 15 the dynamic response of the system to the changes in the set-points is presented. A stochastic signal is superimposed at the output to emulate the effect of turbulence seen in Figure 8 and Figure 9. At *t* = 100 s a negative step input is applied to both set-points, while at *t* = 250 s a positive step input is applied. The figures show that the controller can track both set-points in the positive and negative step changes. Both dynamic responses have a settling time of ~20 s, although the jet velocity overshoots the set-point before settling down. Furthermore, Figure 16 and Figure 17 show the manipulated variables during the experiments. The controller can achieve the control objectives without exceeding the constraints on the manipulated variables. In the end, these optimal conditions promote the optimal double roll pattern by avoiding a deeper impingement into the mold. This allows for the formation of sufficient upper flow circulations as seen in Figure 2 that prevents the entrapment of impurities. Furthermore, it should be noted that the inputs and outputs of this system are coupled as shown in Figure 8 and Figure 9. Both manipulated variables influence both controlled variables. This should be taken in consideration when deciding the values for the set-points for the controller. Moreover, the optimal values for the jet impingement and jet velocity chosen for these experiments have been selected after a careful literature review and detailed analysis of the measured velocity fields from Mini-LIMMCAST. In the future, the ideal way to select these values for industrial application would be to observe the quality of the steel product after solidification to identify the optimum values needed to avoid defects in the steel.

As previously stated in Section 2.2 using flow information extracted in real time for closed loop control is still an open problem and it is not widely addressed in the literature. The only exception is the paper by Dekemele et al. [18], where a mechanical apparatus called submeniscus velocity control device is used to obtain the meniscus velocity at a specific point and an electromagnetic stirrer is used as the actuator. The approach described in the present paper differs from [18] in two significant points. Firstly, we are using a two-dimensional flow map to obtain the information about flow patterns. This gives richer information on flow fields in the mold. The developed procedure can be easily adapted to contactless inductive flow tomography (CIFT), which has the potential to be applied at an industrial caster [29]. Secondly, the feedback control described in [18] is single input single output control. The only controlled variable is the meniscus speed. However, our approach utilizes the velocity profile of the mold and extracts the necessary features to achieve the optimal flow structures. The controller is then designed based on these features.

## 6. Conclusions

Due to the limitations of applying conventional sensors to the flow in the mold of a continuous caster, this paper proposes the use of tomographic sensors to control the flow structure in the mold by obtaining the velocity profile in the mold. In the experiments conducted in this paper, UDV is used as a tomographic modality to obtain the velocity profile in the region surrounding the SEN in the mold. By extracting the necessary features from the velocity fields, a controller based on Model Predictive Control was designed to achieve optimum values for both the impingement point and the velocity of the jet. It was shown that the controller is able to adjust the flow structure in the mold according to the given set points. The techniques used in this paper for control loop design can be utilized for other tomographic sensors if similar information on the velocity fields of the mold is obtained. Based on the results achieved, the following conclusions can be made:

(1) We can avoid using the entire velocity map measured by the UDV by quantifying specific features from the raw data. This allows for less processing time for the controller and allows for conventional control techniques to be applied as the distributed parameter system is simplified to a lumped parameter system.
(2) The deterministic part of the relationship between the manipulated variables (current of the electromagnetic brake and position of the stopper rod) and the controlled variables (jet impingement point and jet velocity) can be described by a model obtained using system identification methods. Measurement data from the experimental setup is used for this process. In the end, a 4th order discrete state space model with two inputs and two outputs can capture the essential dynamics of the relationship between manipulated and controlled variables.
(3) This black-box model created by using system identification is able to describe the dynamic responses of the system, however it should be taken into consideration that the stochastic effect of turbulent flow is not described by the model.
(4) The model is precise enough to be used as a part of the model based predictive controller. The control technique allows the constraints on the manipulated variables to be taken into account while designing the algorithm for the controller. The controller is able to track both set-points without exceeding any constraints. In this way optimal flow structures in the mold can be achieved.

## Figures and Tables

**Table 1:** Physical properties of gallium-indium-tin [17].

	Values
Reference Temperature (°C)	20
Density (ρ)	6353
Kinematic Viscosity (υ)	3.44 × 10^−7^
Electrical Conductivity (σ)	3.29 × 10^−7^
Thermal Conductivity (λ)	23.98
Surface Tension	0.587

**Table 2:** Dimensions of the Mini-LIMMCAST setup [17].

	Dimensions
Mold Width (mm)	300
Mold Thickness (mm)	35
Mold Height (mm)	600
Submerged Entry Nozzle Immersion Depth (mm)	35 ± 10
Submerged Entry Nozzle Inner Diameter (mm)	12
Submerged Entry Nozzle Outer Diameter (mm)	21
Submerged Entry Nozzle Port Width (mm)	11
Submerged Entry Nozzle Port Height (mm)	13
Submerged Entry Nozzle Port Angle (deg)	−15
Electromagnetic Brake Windings per Coil	32
Electromagnetic Brake Max. Current (A)	600
Electromagnetic Brake Max. Magnetic Flux Density (mT)	404

**Table 3:** Model predictive control design parameters.

	Values
Sample Time ($T_{s}$)	0.50
Prediction Horizon (*p*)	10
Control Horizon (*m*)	4
Output Variable Reference Tracking Weight ($w^{y}$)_1_	0.06
MV_1_ Reference Tracking Weight	0
Manipulated Variable Increment Suppression Weight ($w^{\Delta u}$)_1_	1.68
Output Variable Reference Tracking Weight ($w^{y}$)_2_	0.06
Manipulated Variable Increment Suppression Weight ($w^{\Delta u}$)_2_	1.68

## Abbreviations

EMBr	Electromagnetic Brake
GaInSn	Gallium-Indium-Tin
UDV	Ultrasound Doppler Velocimetry
MPC	Model Predictive Control
SEN	Submerged Entry Nozzle
ECT	Electrical Capacitance Tomography
